# Large Spin-Dependent Thermoelectric Effects in NiFe-based Interconnected Nanowire Networks

**DOI:** 10.1186/s11671-020-03343-8

**Published:** 2020-06-29

**Authors:** Nicolas Marchal, Tristan da Câmara Santa Clara Gomes, Flavio Abreu Araujo, Luc Piraux

**Affiliations:** Institute of Condensed Matter and Nanosciences, Université catholique de Louvain, Place Croix du Sud 1, Louvain-la-Neuve, 1348 Belgium

**Keywords:** 3D magnetic nanowire networks, Spintronics, Spin caloritronics, Thermoelectricity, NiFe alloys

## Abstract

NiFe alloy and NiFe/Cu multilayered nanowire (NW) networks were grown using a template-assisted electrochemical synthesis method. The NiFe alloy NW networks exhibit large thermopower, which is largely preserved in the current perpendicular-to-plane geometry of the multilayered NW structure. Giant magneto-thermopower (MTP) effects have been demonstrated in multilayered NiFe/Cu NWs with a value of 25% at 300 K and reaching 60% around 100 K. A large spin-dependent Seebeck coefficient of –12.3 *μ*V/K was obtained at room temperature. The large MTP effects demonstrate a magnetic approach to control thermoelectric properties of flexible devices based on NW networks.

## Introduction

Thermoelectric effects in spintronic materials are actively studied in the emerging field of spin caloritronics due to their unique physical properties including spin Seebeck effects, thermally generated spin current and thermal-assisted spin-transfer torque [1–7]. Also, the thermoelectric analogs of the magnetoresistive effects in magnetic multilayers, spin valves, and tunneling junctions such as the giant magneto-Seebeck and magneto-Peltier effects are of special interest, as they could be used to enable magnetic control of heat flow and thermoelectric voltages for waste-heat recovery from electronic circuits [3, 8–13]. The large spin-dependent thermoelectric effects achieved by modifying appropriately the magnetization configurations of the multilayer with an external magnetic field exploit the fact that the Seebeck coefficients for spin-up and spin-down electrons are significantly different. This difference of Seebeck coefficients is ascribed to the d-band exchange splitting in transition ferromagnetic (FM) metals, as suggested from previous works performed on dilute magnetic alloys [14, 15]. When considering the Peltier effect, it means that different amount of heat is carried by the spin-up and spin-down electrons. It was recently demonstrated that interconnected magnetic nanowire (NW) networks fabricated by electrochemical deposition in 3D nanoporous polymer host films provide an attractive pathway to fabricate light, robust, flexible, and shapeable spin caloritronic devices in versatile formats that meet key requirements for electrical, thermal, and mechanical stability [16, 17]. In addition, electrochemical synthesis is a powerful method for fabricating multicomponent nanowires with different metals due to its engineering simplicity, versatility, and low-cost [18–20]. In such centimeter-scale nanowire networks, electrical connectivity is essential to allow charge flow over the whole sample sizes. The nanowire-based system overcomes the lack of reliability and reproducibility of the results obtained in metallic nanopillars and magnetic tunnel junctions [3, 9, 10, 12], which can be mainly attributed to the thermal contact resistance between the nanoscale samples and the thermal baths which generate the temperature gradient. The 3D nanowire networks hold promise for flexible thermoelectric generators exhibiting extremely large and magnetically modulated thermoelectric power factor. The conventional thermoelectric modules consist of coupled n- and p-type thermoelectric materials or legs. While initial work has focused on n-type NW systems made of Co/Cu and CoNi/Cu multilayers [16, 17], it was recently shown that dilute NiCr alloys are promising for the fabrication of p-type nanowire-based thermoelectric legs [21]. In the present work, we report on experimental results obtained on other n-type thermoelectric films based on interconnected Ni, NiFe alloys, and Ni_80_Fe_20_/Cu multilayered NW networks. Nickel-iron is an important soft magnetic material that is widely used in magnetic data storage technologies. NiFe alloys with optimized sample compositions also exhibit large thermopower near room temperature. In addition, NiFe/Cu multilayers are well-known giant magnetoresistance (GMR) systems [22]. The physical origin of GMR is the different conduction properties of the majority and minority spin electrons in magnetic multilayers. Through magneto-thermopower measurements and exploiting the fact that the branched nanowire architecture of these multilayer NW networks allows electrical measurements in the current perpendicular to the plane (CPP) geometry, a precise determination of spin-dependent Seebeck coefficients in permalloy (Ni_80_Fe_20_) is obtained.

## Experimental Methods

**Fig. 1:**
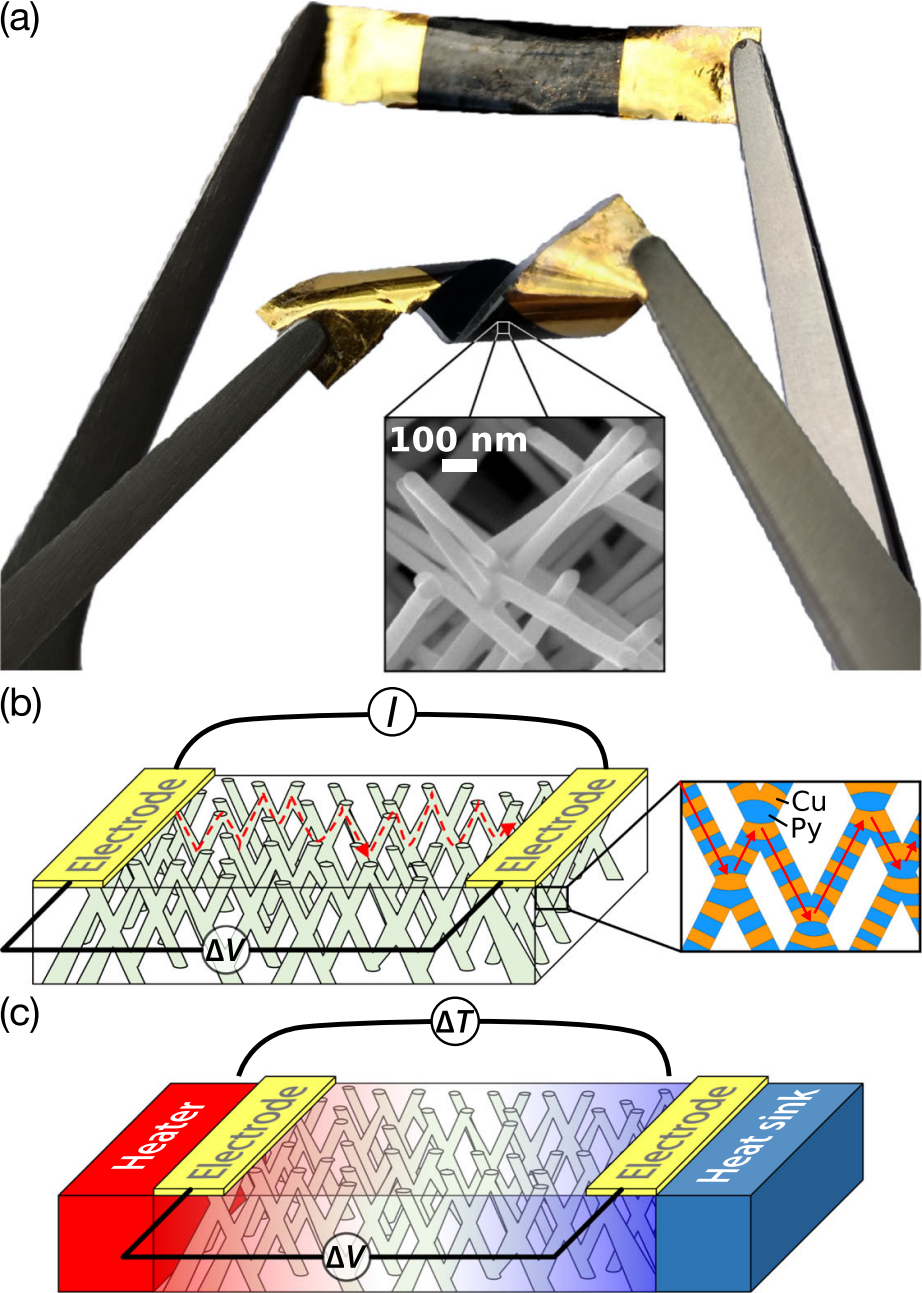
**a** Photograph of a flexible spin caloritronic device based on a nanowire network. The inset SEM image shows the nanowire branched structure with diameter of ∼80 nm. Schematic representation of an electrode design for electrical (**b**) and thermoelectric (**c**) measurements of an interconnected NW network. The inset of Fig. 1b shows a schematic drawing of the Py/Cu multilayer structure. Red arrows represent the direction of current flow. The color in **c** represents the generated temperature profile in the NW networks

The polycarbonate (PC) porous membranes with interconnected pores have been fabricated by exposing a 22- *μ*m-thick PC film to a two-step irradiation process [23, 24]. The topology of the membranes was defined by exposing the film to a first irradiation step at two fixed angles of −25^∘^ and +25^∘^ with respect to the normal axis of the film plane. After rotating the PC film, in the plane by 90^∘^, the second irradiation step took place at the same fixed angular irradiation flux to finally form a 3D nanochannel network. Then, the latent tracks were chemically etched following a previously reported protocol [25] to obtain 3D porous membranes with pores of 80-nm diameter and volumetric porosity of 3%. Next, the PC templates were coated on one side using an e-beam evaporator with a metallic Cr (3 nm)/Au (400 nm) bilayer to serve as cathode during the electrochemical deposition. The NW network partially fills the 3D porous PC membrane. NiFe alloy NWs of controlled composition with Fe content below 40% were successfully grown at room temperature using a sulfate bath and depositing at different potentials [26]. In addition, electrodeposited Py (permalloy, Ni_80_Fe_20_)/Cu multilayered nanowires were made from a single-sulfate bath containing Ni ^2+^, Fe ^2+^, and Cu ^2+^ ions by using a pulsed electrodeposition technique as described in ref. [27]. Following a procedure described elsewhere [18], the deposition rates of each metals were determined from the pore filling time. The thickness of the bilayers was set as 10 nm with approximately the same thickness for the Py and Cu layers. Cu impurity is incorporated only to a very limited content (less than 5%) in permalloy, as evaluated by energy-dispersive X-ray analysis (EDX). The microstructure of single NiFe and NiFe/Cu nanowires grown by electrodeposition in nanopores was previously investigated using X-ray diffraction and analytical transmission electron microscopy [28]. Figure 1a illustrates the flexibility of the spin caloritronic device film based on an interconnected nanowire network. The film can be easily twisted without damaging its electrical properties. The chemical dissolution of the PC template using dichloromethane leads to an interconnected metallic self-standing structure (inset of Fig. 1a) that faithfully replicate the 3D porous template. For conducting electrical and thermoelectric transport measurements, the cathode was locally removed by plasma etching to create a two-probe design suitable for electric measurements as shown in Fig. 1b, c [16, 29, 30]. In this configuration, the current is directly injected to the branched CNW structure (about 1-cm long) from unetched sections of the metallic cathode, where the electrical contacts are directly made by Ag paint, and goes through the 20- *μ*m-thick NW network thanks to the high degree of electrical connectivity of the CNWs. Moreover, since the flow of electrical and thermal currents is restricted along the nanowire segments, the current flows perpendicular to the plane of the layers in the case of a multilayered structure. The typical resistance values of the prepared specimens are in the range of few tens of ohms. For each sample, the input power is kept below 0.1 *μ*W to avoid self-heating, and the resistance was measured within its ohmic resistance range with a resolution of one part in 10^5^. Heat flow is generated by a resistive element and a thermoelectric voltage *ΔV* is created by the temperature difference *ΔT* between the two metallic electrodes. The voltage leads were made of thin Chromel P wires, and the contribution of the leads to the measured thermoelectric power was subtracted out using the recommended values for the absolute thermopower of Chromel P in NIST ITS-90 Thermocouple Database. The temperature gradient was monitored with a small-diameter type E differential thermocouple. A typical temperature difference of 1 K was used in the measurements. For magnetoresistance (MR) and magneto-thermopower (MTP) measurements, the external magnetic field was applied along the out-of-plane (OOP) and in-plane (IP) directions of the NW network films (for more details see thermoelectric measurements and correction factor in the Additional file 1).

## Results and Discussions

**Fig. 2:**
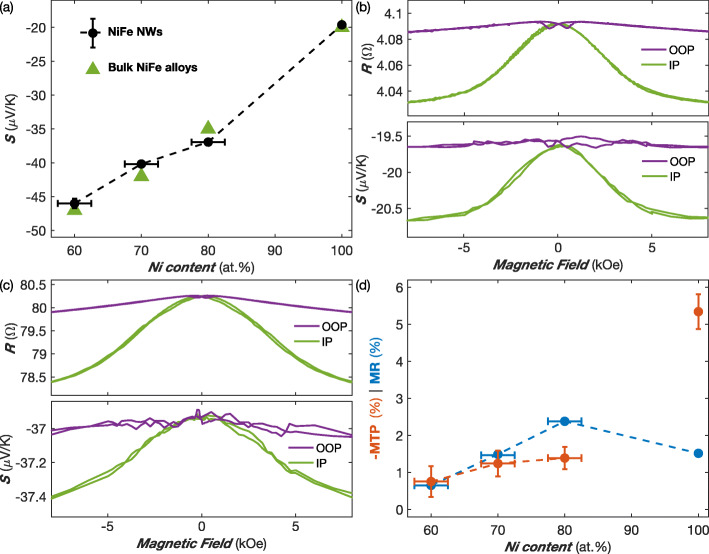
**a** Variation of the Seebeck coefficient vs Ni content in NiFe NW networks (80-nm diameter) at room temperature. Recommended values for bulk alloys [38] are also reported. **b**, **c** Room temperature variation of the electrical resistance and Seebeck coefficient of Ni (**b**) and Py (**c**) NW samples obtained with the applied field in-plane (IP) and out-of-plane (OOP) of the NW network film. **d** MR and MTP ratios as a function of Ni content in NiFe NW networks at RT

The absolute thermoelectric power at room temperature (RT) of pure Ni and NiFe alloy NW networks containing 20%, 30%, and 40% Fe is shown in Fig. 2a. The thermopower increases continuously with increasing Fe content, reaching values between –20 *μ*V/K for pure Ni to about –45 *μ*V/K for Ni_60_Fe_40_. The error bars in Fig. 2a are due to uncertainties in the composition of the alloys related to the electroplating process. These results are in good agreement with the experimental data obtained on bulk NiFe alloys [31]. Therefore, NiFe alloys with fine-tune composition potentially yield significantly larger Seebeck coefficients than pure ferromagnetic metals like Co and thermocouple materials like constantan (Cu_55_Ni_45_: *S*≈ -38 *μ*V/K). We also note that the measured value for Py NWs (*S*≈ -37 *μ*V/K) is very similar to the reported bulk values in the literature [32, 33]. The panels b and c of Fig. 2 show the RT magnetic field dependencies of the resistance and thermopower of Ni and Py NW networks with the field applied in the IP and OOP directions. The resistance and thermopower of the Py and Ni NW samples show the same magnetic field dependencies along the two directions. The *R*(*H*) curves correspond well to the anisotropic magnetoresistance effect, which is due to the anisotropy of spin-orbit scattering in transition ferromagnetic metals. This effect leads to a decrease in resistivity as the angle between the magnetization and current directions is increased. Indeed, the current flow being restricted along the NW segments, the saturation magnetization in the IP direction makes an average angle of ± 65^∘^ with the current. In contrast, when the magnetization is saturated in the OOP direction, the average angle between the magnetization and the current flow is much smaller (±25^∘^). Therefore, the decrease of resistance in an externally applied magnetic field is enhanced when the field is applied in the IP direction. Obviously, the lower resistance state expected for the perpendicular configuration between magnetization and current could not be achieved in such NW networks. The observation that the absolute value of the thermopower increases with increasing transverse magnetic field in both Ni and NiFe alloy NW networks is also in good agreement with previous studies performed on single NWs [34]. Figure 2d shows the magnitude of the magnetoresistance and magneto-thermopower evaluated at RT in the IP direction for pure Ni and NiFe alloy NW networks. Here, the MR and MTP ratios are defined as MR =(*R*(*H*=0)−*R*(*H*_sat_))/*R*(*H*=0) and MTP =(*S*(*H*=0)−*S*(*H*_sat_))/*S*(*H*=0), with *R*(*H*_sat_) and *S*(*H*_sat_) the resistance and thermopower at *H*= 10 kOe, respectively. For the NiFe alloy samples, the magnitude MTP ratio is either comparable or smaller (Py) to the MR ratio. Such smaller value of the MTP ratio with respect to the corresponding MR ratio for the Py NW network is in agreement with measurements performed on Py thin films [35]. In contrast, the Ni NW network exhibits a MTP effect of –5% much larger than the MR ratio of 1.5%. This result is in good agreement with previous measurements performed on single Ni NWs, showing the same enhancement of the MTP effect [34]. It is interesting to note that for Ni thin films, the observed anisotropy of the Seebeck coefficient has approximately the same magnitude than the anisotropic MR (∼1.5%) [35]. Further studies are needed to understand this unexpected enhanced MTP for Ni NWs.

In FM/Cu multilayers, the Seebeck coefficient in the direction perpendicular to the layers can be calculated from the corresponding transport properties using Kirchhoff’s rules [36],

1 $$ S_{\perp} = \frac{S_{\text{Cu}} \kappa_{\text{FM}} + \lambda S_{\text{FM}} \kappa_{\text{Cu}}}{\lambda \kappa_{\text{Cu}} + \kappa_{\text{FM}}},   $$

where *S*_FM,Cu_ and *κ*_FM,Cu_ represent the thermopower and the thermal conductivity of the ferromagnetic material and Cu and *λ*=*t*_FM_/*t*_Cu_ the thickness ratio of FM and Cu layers. According to Eq. 1, *S*_⊥_ is mainly determined by the large thermopower of the FM metal in case the thickness ratio *λ* is not too small since *S*_FM_*κ*_Cu_>>*S*_Cu_*κ*_FM_.

**Fig. 3:**
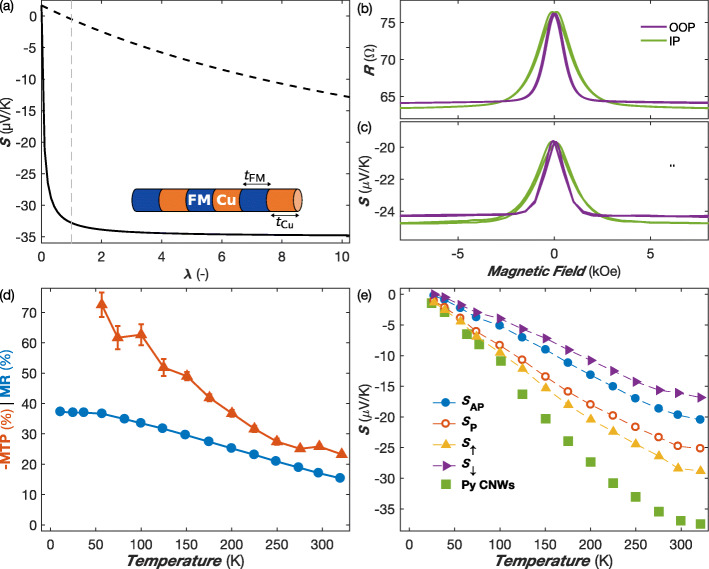
**a** Calculated thermopower for Py/Cu multilayers in the layer parallel (dashed line) and perpendicular (solid line) directions vs thickness ratio *λ*=*t*_Py_/*t*_Cu_ using Eqs. 1 and 2 and bulk values for transport coefficients. The gray dashed line shows the values for *λ*= 1; the inset shows a FM/Cu multilayer stack. **b** Room temperature variation of the electrical resistance and Seebeck coefficient of a Py/Cu NW network in magnetic fields applied in the IP and OOP directions. **c** MR ratio and MTP as a function of temperature with the field applied in the plane of the NW network films. **d** Measured Seebeck coefficients at zero applied field *S*_AP_ (blue full circles) and at saturating magnetic field *S*_P_ (red open circles), along with the corresponding calculated *S*_*↑*_ (orange triangles) and *S*_*↓*_ (violet triangles) from Eqs. 5 and 6 (see text). The data obtained on a Py NW network (80-nm diameter) are also reported (green squares). The error bars reflect the uncertainty of the electrical and temperature measurements and is set to two times the standard deviation, gathering 95% of the data variation

In contrast, the Seebeck coefficient of a FM/Cu multilayer stack in the direction parallel to the layers is given by

2 $$ S_{\parallel} = \frac{S_{\text{Cu}} \rho_{\text{FM}} + \lambda S_{\text{FM}} \rho_{\text{Cu}}}{\lambda \rho_{\text{Cu}} + \rho_{\text{FM}}},   $$

with *ρ*_FM_ and *ρ*_Cu_ as the corresponding electrical resistivities. In this case, large thermopower can be obtained only in case the thickness ratio *λ* is very large. The contrasting behavior between layer parallel and perpendicular directions is illustrated in Fig. 3a for Py/Cu multilayers using Eqs. 1 and 2, and the literature resistivity and thermopower values for bulk permalloy [32, 33, 37, 38] (*ρ*_Py_≈ 25 *μΩ*cm, *S*_Py_= –35 *μ*V/K) and copper (*ρ*_Cu_= 1.6 *μΩ*cm, *S*_Cu_= 1.7 *μ*V/K), as well as the thermal conductivities estimated from the Wiedemann-Franz law (*κρ*=*LT*, where *T* is the temperature and *L* is the Lorenz ratio). For bulk Py single crystal, the relatively small lattice contribution to the thermal conductivity is expected to change slightly the estimated value. Although the electrical resistivity and thermal conductivity values of multilayer nanowires may vary considerably from their respective bulk constituents, the same contrasting behavior between the parallel and perpendicular directions of the layers remains. So, multilayered NWs with alternate stacks of dissimilar materials such as Py and Cu (see Fig. 3a) are promising candidates for good thermoelectric materials.

As shown in Fig. 3b, the resistance and thermopower of the Py/Cu NW network show the same magnetic field dependencies along the OOP and IP directions of the NW network film. The easy axis is pointing along the OOP direction, with a saturation magnetic field of about 1.8 kOe. The sample was found to exhibit large GMR responses (using the current definition of the GMR ratio in which the MR effect is normalized to the lower resistance state R _P_, i.e., GMR =*R*_AP_/*R*_P_−1, with *R*_AP_ and *R*_P_ as the corresponding resistances in the high- and low-resistance states) reaching RT values of 20.5% and 19% along the IP and OOP directions, respectively. The small difference is ascribed to the anisotropic magnetoresistance contribution. As expected, the measured RT thermopower on the CPP-GMR Py/Cu NW network in the saturated state (*S*≈ –25 *μ*V/K along the IP direction) is only slightly smaller than the value found in the homogeneous Py sample. In contrast, the RT Seebeck coefficients reported for NiFe/Cu multilayers in the CIP geometry (∼-10 *μ*V/K) are much smaller [39]. Hereafter, only the measurements obtained in the plane of the NW network films are reported. As shown in Fig. 3c, the absolute value of the magneto-thermopower MTP =(*S*_AP_−*S*_P_)/*S*_AP_, with *S*_AP_ and *S*_P_ the corresponding diffusion thermopowers in the high- and low-resistance states, respectively, increases monotonically with decreasing temperature in a similar manner as the MR ratio (defined as MR =(*R*_AP_−*R*_P_)/*R*_AP_). However, while the magnitude of the effects are similar near RT, the MTP exhibits a pronounced reinforcement in the low temperature range. This behavior is in contrast with what has been observed on Co/Cu and CoNi/Cu NW networks, which exhibit a marked drop in their MTP at low temperatures [16, 17]. Around *T*= 50 K, the MTP reaches about 70% for the Py/Cu sample, which is found to be 2 to 3 times larger than that of Co/Cu and CoNi/Cu NW networks. The GMR ratio at low temperatures (∼60%) is only slightly smaller than the ones previously reported on arrays of parallel Py/Cu NWs [27, 40], thus demonstrating that high-performance CPP-GMR flexible films based on NW networks can be fabricated by this simple and inexpensive bottom-up method.

Using a simple consideration of the parallel current paths of spin-up and spin-down electrons [41], the corresponding thermopowers in the high and low resistance states, S _AP_ and S _P_, are simply given by:

3 $$ S_{\text{AP}} = \frac{S_{\uparrow} \rho_{\uparrow}+ S_{\downarrow} \rho_{\downarrow} }{\rho_{\uparrow} + \rho_{\downarrow}},   $$

and:

4 $$ S_{\mathrm{P}} = \frac{S_{\uparrow} \rho_{\downarrow}+ S_{\downarrow} \rho_{\uparrow} }{\rho_{\uparrow} + \rho_{\downarrow}},   $$

where separate resistivities *ρ*_*↑*_ and *ρ*_*↓*_ and Seebeck coefficients *S*_*↑*_ and *S*_*↓*_ are defined for majority and minority spin channels. Therefore, the spin-dependent Seebeck coefficients, *S*_*↑*_ and *S*_*↓*_ can be expressed as follows [16]:

5 $$ S_{\uparrow} = \frac{1}{2} \big[S_{\text{AP}}\big(1-\beta^{-1}\big) + S_{\mathrm{P}}\big(1+\beta^{-1}\big) \big],   $$

6 $$ S_{\downarrow} = \frac{1}{2} \big[S_{\text{AP}}\big(1+\beta^{-1}\big) + S_{\mathrm{P}}\big(1-\beta^{-1}\big) \big],   $$

where *β*=(*ρ*_*↓*_−*ρ*_*↑*_)/(*ρ*_*↓*_+*ρ*_*↑*_) denotes the spin asymmetry coefficient for resistivity. A rough estimate of *β*= 0.6 at low temperatures using *β*=MR^1/2^ is in reasonable agreement with previous results from the CPP-GMR experiments performed on Py/Cu multilayers [42]. From Eqs. 5 and 6, it can be easily deduced that *S*_*↑*_=*S*_P_ and *S*_*↓*_=*S*_AP_ in the limit of an extremely large MR ratio (*β*→1). Figure 3d shows the temperature evolutions of *S*_AP_, *S*_P_, *S*_*↑*_, and *S*_*↓*_. Below RT, the various Seebeck coefficients decrease almost linearly with decreasing temperature, which is indicative of the dominance of diffusion thermopower. The data obtained on a homogeneous Py NW network are also shown in Fig. 3d for comparison. For permalloy NWs, the magnitude of the Seebeck coefficient is close to that estimated for *S*_*↑*_, as expected from Eq. 4. The RT value for the spin-dependent Seebeck coefficient *ΔS*=*S*_*↑*_−*S*_*↓*_ of –12.3 *μ*V/K in the Py/Cu NW network is larger than the ones previously obtained for Co/Cu and CoNi/Cu NWs [16, 17]. It is also much larger than the ones indirectly estimated from measurements performed on Py/Cu/Py nanopillar and lateral spin devices valve using a 3D finite-element model [3, 11]. In these previous experiments on Py/Cu nanostructures, it was difficult to determine and/or eliminate the contact thermal resistance, a major source of error, and simulations were often necessary to estimate the temperature gradient over the multilayer stacks. The room temperature spin-dependent Seebeck coefficients of different magnetic multilayer systems are summarized in Table 1. In a previous work, it was suggested that infinitely large MTP is expected when the product *βη* tends to –1 [16]. From the above analysis, the product *βη* near RT for Py/Cu nanowires is estimated near –0.1, thus giving rise to similar magnitude of MTP and MR, as shown in Fig. 3d.

**Table 1:** Room temperature spin-dependent Seebeck coefficients of different magnetic multilayer systems

Magnetic multilayer system	*ΔS* (*μ*V/K)
Py/Cu/Py nanopillar [3]	–3.8
Py/Cu/Py lateral spin devices [11]	–4.5
Co lateral spin devices [11]	–1.8
Co/Cu nanowire network [17]	–8.5
CoNi/Cu nanowire network [16]	–10.0
Py/Cu (this work)	–12.3

## Conclusion

In summary, large scale synthesis of uniform Ni, NiFe alloy, and Py/Cu multilayered nanowire networks was made by electrodeposition into 3D porous polymer templates. We found an unexpected high value of 5% for the MTP of Ni NWs compared with that of the MR (∼ 1.5%). The NiFe alloy nanowire networks display large thermopower, up to about – 45 *μ*V/K for Ni_60_Fe_40_ at room temperature. The Py/Cu NWs exhibit giant magnetoresistance and magneto-thermoelectric effects in the current perpendicular-to-plane geometry, which exceeds 50% at low temperatures. We also found a large spin-dependent Seebeck coefficient of –12.3 *μ*V/K at room temperature, which is larger than previously reported values on metallic magnetic multilayers. Thanks to the ease to fabricate geometrically engineered magnetic nanowires and multilayers by electrodeposition, and their excellent electrical and thermoelectric properties, these 3D NW networks exhibit great potential for use as extremely light and flexible spin caloritronic devices. Such effects could be applied, for example, by using and converting the energy of waste heat occurring in electronic devices or conversely to provide active cooling solutions for electronic devices.

## Supplementary information

**Additional file 1** Thermoelectric measurements and correction factor.

## Data Availability

The datasets used and/or analyzed during the current study are available from the corresponding author on reasonable request.
