# Supplementary material for: Large Spin-Dependent Thermoelectric Effects in NiFe-based Interconnected Nanowire Networks
Source: Nanoscale Res Lett. 2020 Jun 29;15:137. doi: 10.1186/s11671-020-03343-8 (PMC7324447; doi:10.1186/s11671-020-03343-8)
Supplement: Supplementary file 1 — Additional file 1 Thermoelectric measurements and correction factor. [file 11671_2020_3343_MOESM1_ESM.pdf]

Additional Files for: Large spin-dependent thermoelectric effects in NiFe-based interconnected nanowire networks

Nicolas Marchal, Tristan da Câmara Santa Clara Gomes, Flavio Abreu Araujo and Luc Piraux*

*Correspondence: luc.piraux@uclouvain.be
Institute of Condensed Matter and Nanosciences, Université catholique de Louvain, Place Croix du Sud 1, 1348 Louvain-la-Neuve, Belgium
Full list of author information is available at the end of the article

Thermoelectric measurements and correction factor

Thermoelectric and magneto-thermoelectric measurements were performed using a home-made set-ups as schematically shown in Figure 1 (c). The thermoelectric power was measured by attaching one end of the sample to the copper sample holder using silver paint and a resistive heater to the other end to generate heat flow. The voltage leads were made of thin Chromel P wires and the contribution of the leads to the measured thermoelectric power was subtracted out using the recommended values for the absolute thermopower of Chromel P S_{Crp}. The temperature gradient ΔT was monitored with a small diameter type-E differential thermocouple. The Seebeck coefficient of the CNW networks were obtained from the measured ΔV at the edges of the Chromel P wire as

$$S_{\mathrm{NW}} = S_{\mathrm{Crp}}(T_{\mathrm{av}}) - \zeta \frac{\Delta V}{\Delta T}, \quad (1)$$

where $(T_{\mathrm{av}}) = (T_{\mathrm{heatsink}} + 0.5\Delta T)$, and ζ is a correction factor related to relative contact positions for the measurements of ΔV and ΔT.

The correction factor ζ has been estimated as the value that minimize the difference between the measured Seebeck coefficient values S_{NW} for different NW networks made of pure metals at room temperature and the expected values S_{ref} as reported in the literature, which yields $\zeta \approx 0.88$. Table 1 provides the Seebeck coefficient values of several NW networks made of pure metals S_{NW} obtained for $\zeta = 0.88$ and $\zeta = 1$ compared with the corresponding reference values S_{ref}. Because the measuring points are averaged at about 2 mm from each other, $\zeta = 0.88$ indicates a difference of about 0.22 mm between the contact positions for the measurements of ΔV and ΔT. In consequence, a correction factor $\zeta = 0.88$ that has been assumed independent of the temperature has been used for all the measurements presented in this work and applied to the results for Co/Cu and CoNi/Cu nanowire network of refs. [16,17].

Table 1 Room temperature measured Seebeck coefficient values S_{NW} of different NW networks made of pure metals using Equation 1 with $\zeta = 1$ and $\zeta = 0.88$, compared to the expected values S_{ref}. All values are expressed in μV/K

Material	$S_{\mathrm{NW}}(\zeta = 1)$	$S_{\mathrm{NW}}(\zeta = 0.88)$	S_{ref}
Py	-45.4	-36.9	-35
Co	-34.8	-28.0	-30
Ni	-25.3	-19.6	-20
Cu	0.7	3.2	1.7
Fe	14.0	15.0	15
